# Clinical Patterns and Follow-Up of Inflammatory Arthritis and Other Immune-Related Adverse Events Induced by Checkpoint Inhibitors. A Multicenter Study

**DOI:** 10.3389/fmed.2022.888377

**Published:** 2022-06-15

**Authors:** José A. Gómez-Puerta, David Lobo-Prat, Carolina Perez-García, Andrés Ponce, Beatriz Frade-sosa, Ana Milena Millán Arciniegas, Fabiola Ojeda, Virginia Ruiz-Esquide, Hector Corominas

**Affiliations:** ^1^Rheumatology Department, Hospital Clinic, Institut d'Investigacions Biomèdiques August Pi i Sunyer (IDIBAPS), Barcelona, Spain; ^2^Department of Medicine, Universitat de Barcelona, Barcelona, Spain; ^3^Rheumatology Department, Hospital de la Santa Creu i Sant Pau, Barcelona, Spain; ^4^Rheumatology Department, Hospital del Mar, Barcelona, Spain

**Keywords:** immunotherapy, adverse (side) effects, checkpoint, arthritis, polymyalgia rheumatica

## Abstract

**Objectives:**

To describe different clinical patterns of rheumatic immune-related adverse events (irAEs) induced by immune checkpoint inhibitors (ICI) and their rheumatic and oncologic outcomes.

**Methods:**

We classified clinical syndromes according to five different categories: non-inflammatory arthralgias (NIA), rheumatoid arthritis (RA)-like, psoriatic arthritis (PsA)-like, polymyalgia rheumatica (PMR)-like, and a miscellaneous group of patients with other syndromes. We conducted a baseline visit and then follow-up in order to determine their clinical pattern, treatment response, and outcome.

**Results:**

We included 73 patients (64% male) with a mean age of 66.1 ± 11.6 years. Main underlying diagnosis was lung carcinoma in 29 (39%) patients, melanoma in 20 (27%), and renal-urothelial cancer in 11 (15%). Main ICI included Pembrolizumab in 24 (32%), Nivolumab 17 (23%), and Atezolizumab 7 (9 %). Seventeen out of seventy-three patients had an underlying rheumatic disease before ICI treatment. Fourteen patients developed other irAEs before or simultaneously with rheumatic syndromes. Main rheumatic irAEs included: RA-like in 31 (42.4%), NIA in 19 (26.0%), PMR-like in 10 (13.7%), and PsA-like in 5 (6.8%), among others. Median time from ICI to irAEs was 5 months (IQR 3–9). Those patients who received combined therapy, had a trend for an earlier presentation than those who received monotherapy (4.3 months IQR 1.85–17 vs. 6 months IQR 3–9.25, *p* = NS). Mean follow-up time was 14.0 ± 10.8 (SD, months). At the last visit, 47 % were taking glucocorticoids and 11% DMARD therapy. At the last visit, 13 (17.8%) patients remained with persistent arthritis, 19 (26%) had intermittent flares, and 39 (53.4%) had a self-limited pattern. Only in 15.1% of patients ICI therapy was discontinued.

**Conclusions:**

We described different patterns according to treatment and irAEs. Combined ICI therapy had an earlier onset of symptoms. Patients who presented as RA-like, had a higher risk of persistent arthritis. After a mean follow-up of more than 1 year, one-fifth of the patients remained with persistent arthritis and 11% required DMARD therapy.

## Introduction

The appearance of immune checkpoint inhibitors (ICI), including anti- cytotoxic T lymphocyte antigen-4 (anti-CTLA-4), and programmed cell death-1 (PD-1)/programmed death-ligand 1 (PD-L1) monoclonal antibodies, constitutes one of the major medical breakthroughs in oncology treatment, leading to impressive clinical results in different types of cancer (1).

However, ICI might induce several side effects such as immune-related adverse events (irAEs) that can add important morbidity and disability in oncologic patients. There is an increase in the number of reports of rheumatic irAEs, due to an increased usage of ICI as well as a better recognition of these new associations. Overall, they occurred in around 5–10% of patients receiving ICI according to some recent reports (2–5), but sometimes many symptoms such as arthralgias, myalgias, fatigue, or sicca symptoms are underreported and/or misclassified in randomized clinical trials as musculoskeletal disorders. The spectrum of irAEs includes a wide range of immune-mediated syndromes. ICI not only can induce inflammatory arthritis but also systemic syndromes such as vasculitis (6), sarcoidosis-like (7), myositis with or without myocarditis (8) and less frequently described cases of systemic lupus erythematosus (9), fasciitis (10), systemic sclerosis (11), antiphospholipid syndrome (12), and dermatomyositis (13) among others.

Knowledge of different rheumatic irAEs induced by ICI has increased in recent years and general recommendations for treatment and follow-up have been developed for several academic societies (14, 15), however clinical patterns, time to onset of different irAEs according to treatment and follow-up are less well-known.

We aimed to describe the main clinical patterns with emphasis on articular involvement, as well as the clinical course and prognosis of a series of rheumatic irAEs from three University centers in Barcelona.

## Materials and Methods

We conducted an observational study including all adult patients referred to the Rheumatology Departments in three University centers in Barcelona (Hospital Clinic, Hospital de la Santa Creu i Sant Pau, and Hospital del Mar) due to the onset of rheumatic syndromes related to ICI treatment. The study period included patients who visited from January 2015 to January 2021. Data collected included demographic features, history of rheumatic diseases as well as ICI indication and type, presence of non-rheumatic irAEs, disease manifestations at irAE onset, and treatment. The diagnostic and treatment approach was done according to clinical judgment in the daily clinical practice setting. Any type of tumors or hematologic neoplasms were included. In order to avoid potential side effects due to chemotherapy (CT), patients who received combined therapy with CT plus ICI were excluded.

We classified clinical syndromes according to 5 different categories: (a) non-inflammatory arthralgia (NIA), (b) rheumatoid arthritis (RA)-like, (c) psoriatic arthritis (PsA)-like in patients with oligo/polyarthritis with or without enthesitis and tenosynovitis, (d) polymyalgia rheumatica (PMR)—like in patients with inflammatory proximal muscle pain with or without arthritis, and (e) a miscellaneous group of patients with other syndromes including sicca syndrome, sarcoidosis, cutaneous or systemic vasculitis, or others.

Since there are no well-established protocols for the treatment of irAE rheumatic syndromes, treatment decisions were based on oncologists' and rheumatologists' clinical judgment and following the current guidelines for the treatment of rheumatic irAEs (14).

### Statistical Analysis

Descriptive statistics were reported as the mean ± standard deviation for normal continuous variables, median and interquartile range (IQR) for non-normal continuous variables, and frequency and proportions for categorical variables. In bivariate analyses, differences in demographics, type of neoplasia and treatment with the presence of persistent arthritis as primary outcome were compared using chi square tests or Fisher's exact tests for categorical variables and Mann-Whitney *U*-tests for ordinal or continuous variables.

We analyzed differences according to the type of treatment and initiation of rheumatic irAEs, as well as differences in appearance according to different syndromes. Also, we analyzed according to ICI monotherapy or combined treatment. Kaplan-Meier time-to-event analysis was used to determine the risk for persistent arthritis during the follow-up excluding patients with previous rheumatic disease.

The statistical analysis was made using SPSS software (IBM SPSS version 23.0, Inc., Chicago, IL, USA) and GraphPad Prism version 7.0 (GraphPad Software, San Diego, CA, USA). All patients gave written informed consent. The study was approved first by the Hospital Clinic Institutional Review Board (HCB/2021/0901) and therefore in the other two Ethics Committee.

## Results

A total of 73 patients were included. Most of the patients were male (64%) and the mean age at the moment of inclusion was 66.1 ± 11.6 years. Main underlying diagnoses for ICI treatment were lung carcinoma in 29 (39.7) cases, melanoma in 20 (27.3%), renal-urothelial cancer in 11 (15%), liver carcinoma in 2 (2.7%), breast cancer in 2 (2.7%), acute myeloid leukemia in 2 (2.7%), and head and neck carcinoma in 2 (2.7%) among others.

Fifty five (75.0%) patients received another treatment for their oncologic diseases. A total of 34 (46.0%) received CT, 10 (13.0%) radiotherapy and 9 (12.3%) surgery. Details for each patient are collected in Supplementary Table 1.

Only 16 (21.9%) out of 73 patients had any rheumatologic disease before ICI, but only 9 had an underlying inflammatory disorder including RA in 3 (4.1%) patients, spondyloarthropathies in 2 (3.2%) patients and two patients with PsA (3.2%) (Table 1), among others. Sixteen (21.9%) out of 73 patients had 19 non-rheumatic irAEs before or simultaneously with the appearance of rheumatic syndrome, and three cases after rheumatic irAEs. Those non-rheumatic irAE syndromes included: colitis in 5 (8%), hypothyroidism in 3 (4.1%), and peripheral neuropathy in 2 (2.7%), among others (Table 2). Mean time from ICI initiation and non-rheumatic irAE was/ 7.65 ± 7.60 months.

**Table 1:** General characteristics, type of cancer and ICI molecules.

	***N*** **= 73 (%)**
Gender (Male)	46 (64.4)
Mean current age (years ± SD)	66.1 ± 11.6
Mean time from CPI initiation and irAE onset (months ± SD)	7.7 ± 7.9
**Type of cancer**	*N* (%)
Lung	29 (39.7)
Melanoma	20 (27.3)
Renal-urothelial	11 (15.0)
Liver	2 (2.7)
Breast	2 (2.7)
Acute myeloid leukemia	2 (2.7)
Head and neck	2 (2.7)
Ovarium	1 (1.3)
Rectum	1 (1.3)
Thyroid	1 (1.3)
Skin	1 (1.3)
Myelodisplastic syndrome	1 (1.3)
**Previous rheumatic/inflammatory diseases**	
None	57 (78.1)
Rheumatoid arthritis	3 (5.4)
Gout	3 (4.1)
Spondyloarthritis	2 (3.2)
Chondrocalcinosis	2 (2.7)
Psoriasis	2 (2.7)
Systemic lupus erythematosus	1 (1.4)
Cryoglobulinemia	1 (1.4)
Fibromyalgia	1 (1.4)
De Quervain tendinitis	1 (1.4)
**Type of Checkpoint inhibitors**	
**Monotherapy**	*N* = 60 (82.2)
Pembrolizumab	24 (32.9)
Nivolumab	17 (23.3)
Atezolizumab	7 (9.6)
Durvalumab	7 (9.6)
Anti-TIM3	3 (4.1)
Avelumab	1 (1.4)
Ipilimumab	1 (1.4)
**Combined therapy**	*N* = 13 (17.8)
Nivolumab + Ipilimumab	8 (11.0)
Pembrolizumab + Epacadostat	2 (2.7)
Durvalumab + Tremelimumab	1 (1.4)
Pembrolizumab + Eftilagimod	1 (1.4)
Ibatasertib + Atezolizumab	1 (1.4)

*Anti-TIM3, Anti T cell immunoglobulin mucin domain 3*.

**Table 2:** Main irAEs non rheumatic features.

**Non-rheumatic irAEs**	***N*** **(%)**
None	57 (78.0)
Colitis	5 (6.8)
Hypothyroidism	5 (6.8)
Peripheral neuropathy	2 (2.7)
Hypophysitis	1 (1.3)
Sarcoidosis	1 (1.3)
Sweet syndrome	1 (1.3)
Vitiligo	1 (1.3)
Interstitial nephritis	1 (1.3%)
Pneumonitis	1 (1.3%)
Hepatitis	1 (1.3%)

*Some patients presented more than 1 non-rheumatic irAE*.

*In 3 patients, non-rheumatic irAE presented after rheumatic irAE*.

### Clinical Presentation

Most of the patients complained of articular or periarticular symptoms including generalized arthralgias in 25 (34.2%), polyarthritis in 12 (16.4%), proximal weakness, pain and stiffness in the shoulders and hips in 11 (15.0%), monoarthritis in 9 (12.3%), oligoarthritis in 7 (9.5%), tenosynovitis in 4 (5.4%), paresthesias in 2 (2.7%), and purpura, symmetrical synovitis with pitting edema, and subcutaneous nodules in one case each.

Finally patients were labeled according to clinical judgment with the following diagnosis: RA-like in 31 (42.4%), PMR in 10 (13.7%), PsA-like in 5 (6.8%), seronegative tenosynovitis in 3 (4.1%), vasculitis in 2 (2.7%), gout in 2 (2.7%), and sarcoidosis in 1 (1.4%). Nineteen patients (26.0%) were classified as NIA.

**Figure 1:**
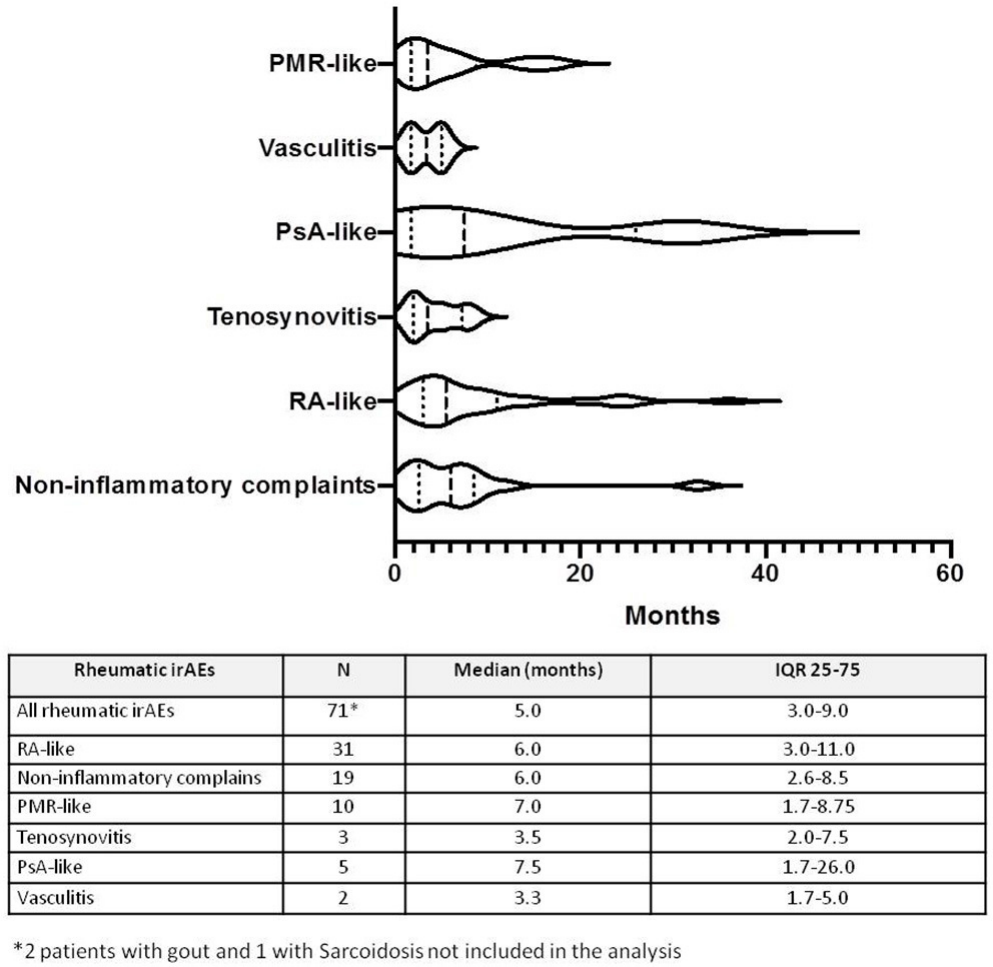
Time to onset from ICI initiation and Rheumatic irAEs according rheumatic diagnosis.

Median time from ICI treatment and irAE onset was 5 months (IQR 3–9 months). Differences according to time of onset and rheumatic irAE are presented in Figure 1. We found some patterns of earlier presentation in those with vasculitis [median time 2 months (IQR 1.7–5.0)] and tenosynovitis [median time 3.5 months (IQR 2.0–7.5)] while a later presentation was found in those patients with RA-like [median time 6 months (IQR 3.0–11.0)], PsA-like [median time 7.5 months (IQR 1.7–26)], and PMR-like [median time 7 months (IQR 1–14)] patterns.

Autoimmune assessment revealed positivity for antinuclear antibody (ANA) (≥1/80) in 24 out of 72 (33%) patients, but only in 6 (8.2%) cases was ANA > 1/320; rheumatoid factor was observed in 5 out of 73 (6.8%) and anti-CCP in 4 out of 71 (5.6%) patients.

Three cases of irAEs induced by Anti T cell immunoglobulin mucin domain 3 (anti-TIM3) deserve special attention. Clinical characteristics of those patients are presented in Table 3 and discussed later.

**Table 3:** Clinical characteristics of patients treated with Anti-TIM3.

**Case**	**Age/**	**Underlying disease**	**Previous Rheumatic/**	**Other irAEs**	**Presenting**	**irAEs Rheumatic**
	**Gender**		**immuno-mediated disease**		**symptom**	**syndrome**
1	72/Male	Acute myeloid leukemia	Uveitis B27 positive	None	Knee monoarthritis	UA
2	72/Male	Acute myeloid leukemia	Psoriasis	Sweet syndrome	Knee monoarthritis	PsA
3	75/Male	Myelodysplastic syndrome	None	None	Tenosynovitis	Tenosynovitis

*UA, Undifferentiated arthritis; PsA, Psoriatic arthritis; TIM3, T cell immunoglobulin and mucin domain 3*.

**Figure 2:**
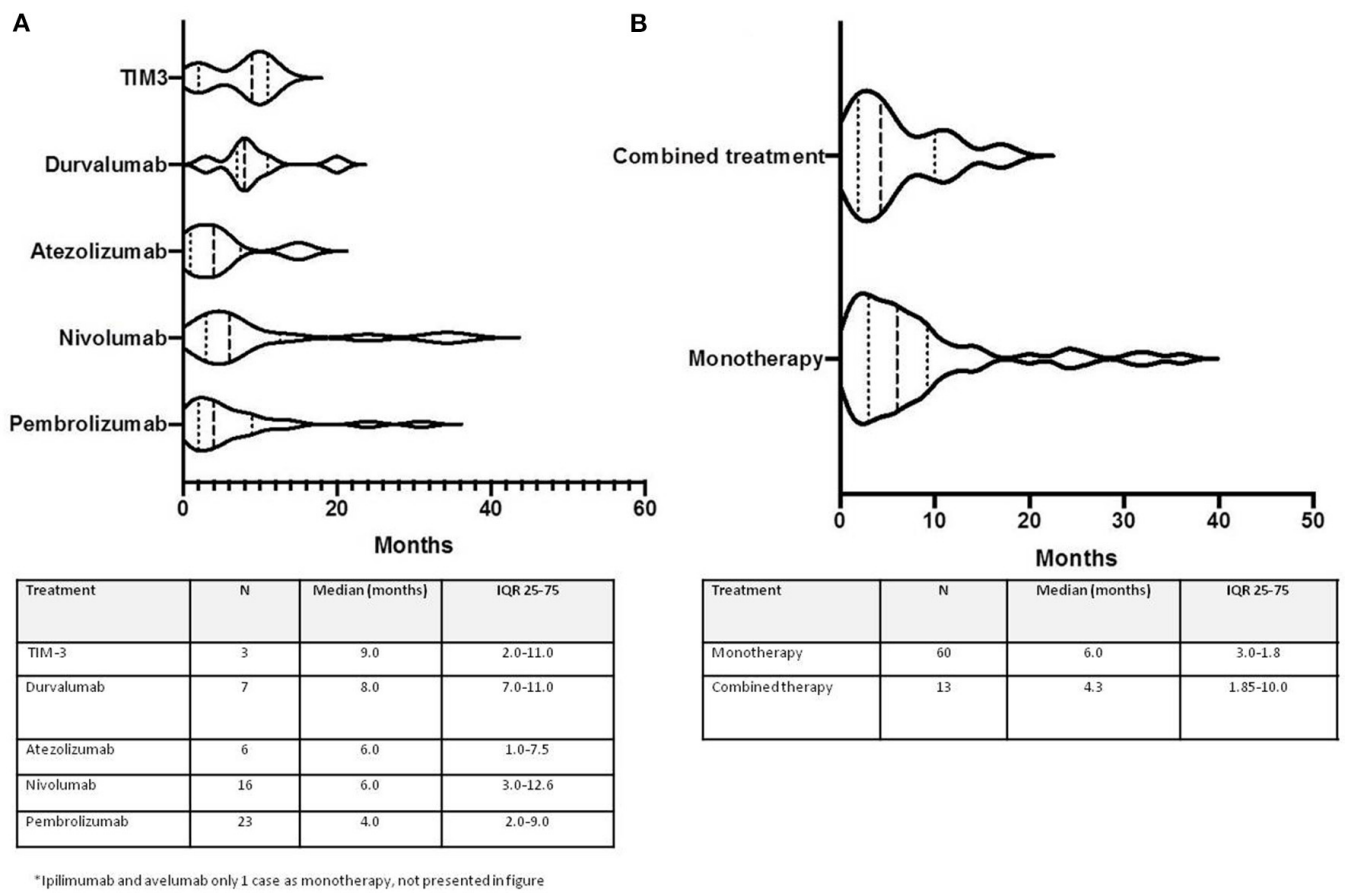
Time to onset from ICI initiation and Rheumatic irAEs according treatment. **(A)** Type of treatment; **(B)** Monotherapy vs. combined.

Time (in months) from ICI initiation to irAE onset was different according to treatments (Figure 2A). For Pembrolizumab, 4 months (IQR 2–9), Nivolumab 6 months (IQR 3–12.6), Atezolizumab 6 months (IQR 1–7.5), Durvalumab 8 months (IQR 7–11), and anti-TIM3 9 months (IQR 2–11).

Those patients who received combined therapy, had a trend for an earlier presentation than those who received monotherapy (4.3 months IQR 1.85–17 vs. 6 months IQR 3–9.25, *p* = NS (Figure 2B).

### Treatment

Rheumatic syndromes were treated according to American Society of Clinical Oncology guidelines including NSAIDs in 23 (31%) patients, glucocorticoids ever used in 44 (60.0%) patients, and currently used in 35 (47.7%) patients with a mean dose of 4.4 ± 7.5 mg per day, and disease-modifying anti-rheumatic drugs (DMARDs) in 8 (11.0%) patients (hydroxychloroquine in four, methotrexate in three, and sulfasalazine in one). Four (5.5%) patients received intra-articular glucocorticoid treatment. In 59 (80.8%) patients, no discontinuation of ICI was needed, in 3 (4.1%) patients combined treatment was changed to monotherapy, and in 11 (15.1%) ICI therapy was discontinued.

### Outcome and Follow-Up

Mean time follow-up was 14.0 ± 10.8 (months). Two patients were lost to follow-up. At the last visit, 13 (17.8%) patients remained with persistent arthritis, 19 (26.0%) had intermittent flares, and 39 (53.4%) had a self-limited pattern. Persistent arthritis was statistically significantly more common in male than female patients (69.2 vs. 30.8%, *p* = 0.018).

**Figure 3:**
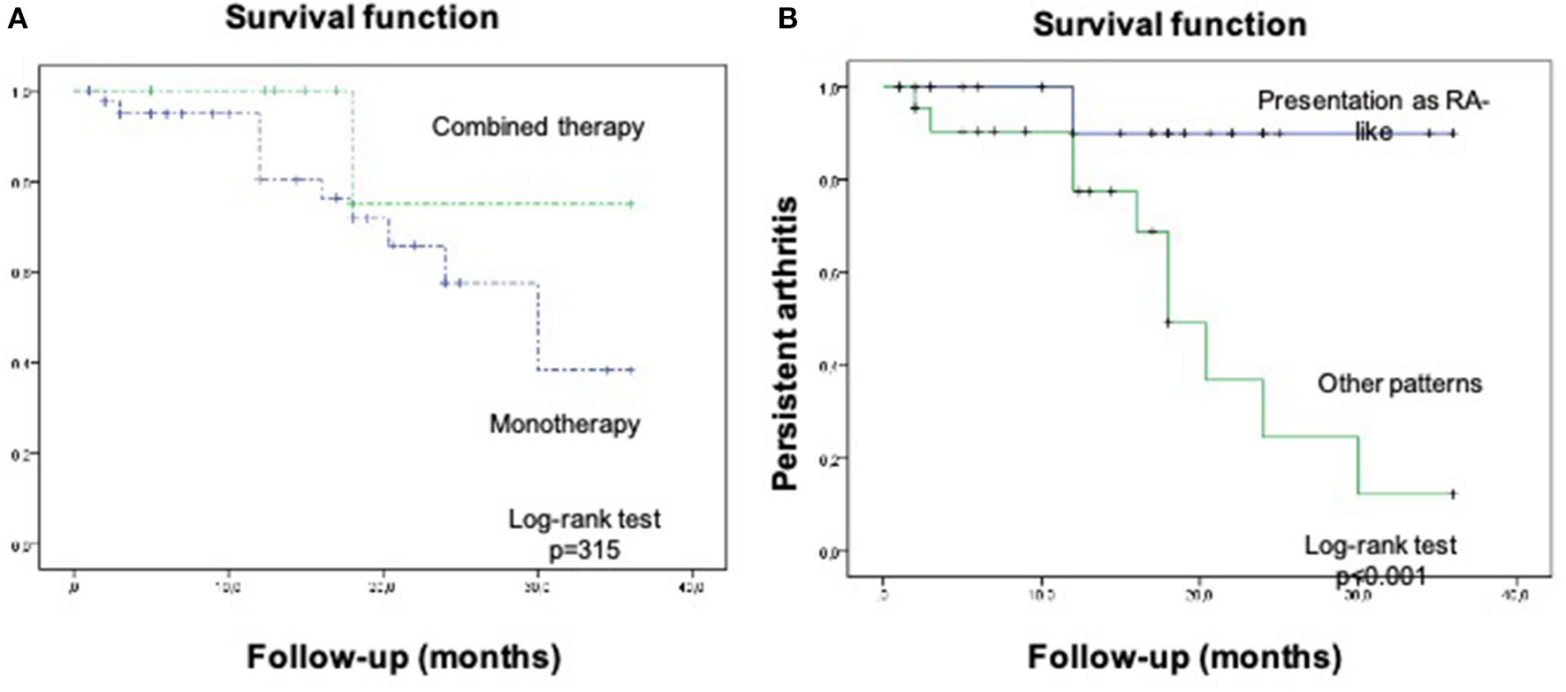
Persistent arthritis in **(A)** patients with monotherapy vs. combined therapy and **(B)** patients presented as RA-Like pattern vs. other patterns.

Patients who had the RA-like pattern had a significantly higher risk for persistent arthritis (Figure 3). No differences were found in the percentage of persistent arthritis in patients with or without previous rheumatic disease, neither with the type of cancer or type of ICI treatment (monotherapy or combined therapy). No differences in the proportion of patients in arthritis remission in patients with or without current glucocorticoid (GC) use were observed.

Regarding oncologic outcomes, 20 (27.4%) patients were in remission, 24 (39.4%) in partial response, and 27 (37.0%) with tumor progression. Fifteen (20.5%) patients died.

## Discussion

We reported clinical patterns and follow-up of rheumatic irAEs from three referral centers. RA-like, PsA, PMR-like, and non-inflammatory complaints were the main clinical phenotype identified in our cohort. After at least 1 year of follow-up, more than half of patients had a self-limited flare, 26% had an intermittent course with flares and remission, and 18% had a chronic persistent pattern as occurs in inflammatory arthritis.

Around 500 patients have been reported in different cohorts of patients mainly from the USA, Canada, France, and Spain (Supplementary Table 2) (2, 3, 5, 10, 16–20). According to previous published data, male patients were the majority (53%), with a mean age of 62.8 ± 3.8 years, and 23% of patients were under DMARD treatment.

Information about predictors of rheumatic irAEs in patients exposed to ICI treatment without previous autoimmune diseases is limited. One study evaluated the presence of HLA class II shared epitope (SE) alleles in 27 patients who developed irAEs and in 299 controls. 61.5% of patients with inflammatory arthritis induced by ICI had at least one SE allele vs. 41.2% of controls (16). A very recent study (21) reported that those patients with melanoma, genitourinary cancer, with pre-existing non-rheumatic autoimmune diseases and who received combination therapy were predictors for the development of rheumatic irAE.

Kostine and colleagues described four different forms of presentation in a series of 35 patients with rheumatic irAEs from 524 patients treated with ICI in a single center (3). Those patterns included PMR, RA, PsA among inflammatory arthritis, and one group with non-inflammatory complaints. Interestingly enough, patients who develop rheumatic irAEs had higher tumor responses than those patients without irAEs suggesting irAEs are predictive of a positive anti-tumor response (22).

To the best of our knowledge, we described here for the first time three patients who developed rheumatic irAE after anti-TIM3 treatment. The immune checkpoint protein TIM3 is a negative regulator of antitumor immunity. TIM3 has a suppressing role of antitumor immunity upon interaction with different ligands such as Galectin 9 (Gal9) (23). Blockade of TIM3/Gal9 is a novel therapeutic approach for hematological myeloid malignancies and solid tumors such as breast and prostate cancer (24).

Few studies have explored the timing of the appearance of the different rheumatic syndromes and the differences according to the treatment used. It is well-known that irAEs are more often seen in patients exposed to combination therapy (22). We observed that rheumatic irAEs appeared earlier in patients with combined therapy, this phenomenon was also described for inflammatory arthralgias by Buder- Bakhaya and colleagues (25). Additionally, in our cohort, patients who received Pembrolizumab, Nivolumab, and Atezolizumab had an earlier onset of rheumatic irAEs. This data should be considered with caution given the small number of cases in some types of treatments. In a John Hopkins cohort (26), among 30 patients with rheumatic irAEs, inflammatory arthritis presented after a median time of 5 months, but no information according to type of treatment or type of rheumatic diagnosis is given.

We found that those patients who presented vasculitis and pure tenosynovitis had an earlier time to onset than patients with RA-like, PMR, PsA-like, or NIA. A recent review of rheumatic irAEs collecting previous reported cases analyzed time to symptom onset after ICI initiation and described a similar pattern, except for earlier presentation in PsA-like cases (27).

The follow-up and clinical course of patients with inflammatory arthritis induced by ICI are less well-known. Almost one out of five patients in our cohort had a persistent arthritis pattern, which has been more common in male patients and in patients with the RA-like pattern, excluding patients with previous rheumatic disease. CanRIO is a Canadian registry that included adult oncologic patients treated with either CTLA-4, PD-1, or PD-L1 in 10 different centers. CanRIO reported new onset inflammatory arthritis in 45 patients, being persistent in 42.0% of them (26). In another cohort of 60 patients followed during a median of 9 months, persistent arthritis was related with combined ICI therapy, longer ICI exposure and prior development of other irAEs (28).

Only 11.0% of our patients required DMARD treatment, and none received biologic DMARDs. Treatment of rheumatic irAE is a challenge for clinicians, including rheumatologists and oncologists. The balance between risk and benefits should be individualized according to oncologic prognosis, ICI response, and severity of the irAE (29). The clinical scenario differs from the “classic” pattern that we usually see in inflammatory chronic arthritis and systemic rheumatic diseases, and many patients had a milder course with fewer requirements for GC or DMARDs (17). Myositis cases could be severe and might suggest a life-threatening situation and require intravenous pulses of glucocorticoids and intravenous immunoglobulins among others (15). Some authors suggest a change in the paradigm for the treatment of rheumatic irAEs, pivoting to a more aggressive approach with early use of biologic DMARDs similar to what occurs in patients with colitis induced by ICI (30). Experience with biologic DMARDs is limited, but some cases reported satisfactory responses with IL-6 inhibitors or TNF-α inhibitors without a significant impact on tumor response (17, 18, 31, 32).

Around one fifth of our cohort had a previous rheumatic disease. We did not find higher rates of persistent arthritis in patients with previous rheumatic disease. In CanRIO (33) they reported irAEs in 27 patients with preexisting autoimmune disease mainly RA, psoriasis/PsA, and inflammatory bowel disease among others. Around half of the patients developed flares of underlying disease, mostly mild, and one-third of the patients had a severe flare that led to ICI discontinuation.

Limitations of the current study include the retrospective nature of the analysis and selection bias of the study cohort since only symptomatic patients were referred to Rheumatology departments. Additionally, a standardized protocol was not followed as the study was conducted in real-world practice. However, we also have strengths, including a relatively big cohort of patients with a clinical follow-up for more than 1 year and the inclusion of new ICI such as anti-TIM3.

Several questions remain unanswered in the field of rheumatic irAEs including DMARD treatment duration, the impact of immunosuppressive drugs over oncologic prognosis, as well as the identification of more predictors for the development of irAE, including rheumatic and non-rheumatic syndromes.

Rheumatic irAEs are still an open scenario and further studies will be needed including new ICI, new combinations of anti-CTLA-4 plus anti-PD1 or anti-PD-L1, and studies with longer follow-up.

## Data Availability Statement

The raw data supporting the conclusions of this article will be made available after request by the authors, without undue reservation.

## Ethics Statement

The studies involving human participants were reviewed and approved by Hospital Clinic Institutional Review Board (HCB/2021/0901). The patients/participants provided their written informed consent to participate in this study.

## Author Contributions

JG-P, HC, and CP-G had access to the study data, developed the figures and tables, wrote the first draft of the manuscript, and vouch for the data and analyses. AP, BF-S, JG-P, HC, CP-G, DL-P, FO, AM, and VR-E contributed to study design, data collection, and patient's follow-up.

## Conflict of Interest

The authors declare that the research was conducted in the absence of any commercial or financial relationships that could be construed as a potential conflict of interest.

## Publisher's Note

All claims expressed in this article are solely those of the authors and do not necessarily represent those of their affiliated organizations, or those of the publisher, the editors and the reviewers. Any product that may be evaluated in this article, or claim that may be made by its manufacturer, is not guaranteed or endorsed by the publisher.

## Abbreviations

ANA: antinuclear antibody
Anti-CTLA-4: anti-cytotoxic T lymphocyte antigen-4
CT: chemotherapy
DMARD: Disease-modifying anti-rheumatic drugs
ICI: Immune checkpoint inhibitors
irAEs: Immune-related adverse events
IQR: Interquartile range
NIA: non-inflammatory arthralgia
PD-1: Programmed cell death-1
PD-L1: Programmed death-ligand 1
PMR: Polymyalgia rheumatic
PsA: Psoriatic arthritis
RA: Rheumatoid arthritis
SE: Shared epitope
UA: undifferentiated arthritis.
