# Supplementary material for: Clinical Patterns and Follow-Up of Inflammatory Arthritis and Other Immune-Related Adverse Events Induced by Checkpoint Inhibitors. A Multicenter Study
Source: Front Med (Lausanne). 2022 Jun 15;9:888377. doi: 10.3389/fmed.2022.888377 (PMC9240301; doi:10.3389/fmed.2022.888377)
Supplement: Supplementary file 1 [file Table_1.docx]

**Supplementary Table 1. General characteristics of a multicenter cohort**

| **Case** | **Age/Sex** | **Type of**  **Neoplasia** | **Previous Rheumatic disease** | **Previous**  **irAEs** | **Type of**  **ICI** | **Previous Oncologic**  **treatment** | **Chemotherapy** | **Radiotherapy** | **Surgical resection** |
| --- | --- | --- | --- | --- | --- | --- | --- | --- | --- |
| 1 | 74/M | LUNG | None | None | Pembrolizumab | No | No | No | No |
| 2 | 72/F | MELANOMA | OA | None | Nivolumab | No | No | No | No |
| 3 | 72/F | LUNG | None | None | Pembrolizumab | No | No | No | No |
| 4 | 59/F | LUNG | SLE | None | Nivolumab | Yes | Yes | No | No |
| 5 | 76/F | LUNG | None | None | Atezolizumab | No | No | No | No |
| 6 | 65/F | HEAD AND NECK | De Quervain Tenosynovitis | None | Nivolumab | Yes | Yes | No | No |
| 7 | 77/M | UROTHELIAL | Chondrocalcinosis | None | Pembrolizumab | Yes | Yes | No | No |
| 8 | 69/M | MELANOMA | None | None | Pembrolizumab | No | No | No | No |
| 9 | 71/F | BREAST | None | None | ibatasertib + Atezolizumab | Yes | No | No | Yes |
| 10 | 65/M | LUNG | None | None | Pembrolizumab | Yes | No | Yes | No |
| 11 | 71/M | MELANOMA | Psoriasis | None | Nivolumab + Ipilimumab | No | No | No | No |
| 12 | 32/F | SQUAMOUS SKIN CANCER | None | None | Pembrolizumab | No | No | No | No |
| 13 | 83/M | MELANOMA | Spondyloarthritis | None | Pembrolizumab | No | No | No | No |
| 14 | 74/M | LUNG | Chondrocalcinosis | None | Atezolizumab | Yes | Yes | Yes | No |
| 15 | 56/M | UROTHELIAL | Gout | None | Nivolumab + Ipilimumab | Yes | No | No | No |
| 16 | 33/F | MELANOMA | None | Colitis | Nivolumab+ Ipilimumab | Yes | Yes | Yes | No |
| 17 | 72/M | ACUTE MIELOID LEUKEMIA | Uveitis B27 | None | Anti TIM3 | Yes | Yes | No | No |
| 18 | 72/M | LUNG | None | None | Nivolumab | Yes | No | No | Yes |
| 19 | 47/M | MELANOMA | None | Polyneuropathy | Ipilimumab | Ye | No | No | No |
| 20 | 53/F | MELANOMA | None | None | Pembrolizumab | No | No | No | No |
| 21 | 51/M | MELANOMA | None | Hypothyroidism | Ipilimumab + Nivolumab | Yes | No | No | No |
| 22 | 55/M | LUNG | None | None | Atezolizumab | Yes | No | No | No |
| 23 | 68/F | MELANOMA | Fibromyalgia | None | Pembrolizumab + Epacadostat | No | No | No | No |
| 24 | 56/F | MELANOMA | RA | Colitis | Pembrolizumab | Yes | Yes | No | No |
| 25 | 65/F | MELANOMA | None | None | Pembrolizumab + Epacadostat | No | No | No | No |
| 26 | 54/M | LUNG | None | None | Durvalumab | Yes | Yes | Yes | No |
| 27 | 59/M | MELANOMA | None | Sarcoidosis | Nivolumab | No | No | No | No |
| 28 | 78/M | UROTHELIAL | None | None | Pembrolizumab | Yes | Yes | No | No |
| 29 | 72/M | ACUTE MYELOBLASTIC LEUKEMIA | Psoriasis | Sweet´s Syndrome | Anti TIM3 | Yes | Yes | No | No |
| 30 | 68/M | MELANOMA | None | None | Pembrolizumab | No | No | No | No |
| 31 | 82/M | LIVER | None | None | Nivolumab | Yes | Yes | Yes | No |
| 32 | 61/M | UROTHELIAL BLADDER | None | None | Durvalumab | No | No | No | No |
| 33 | 76/M | MELANOMA | None | Hypophysitis, Thyroiditis | Ipilumumab + Nivolumab | No | No | No | No |
| 34 | 61/M | LUNG | Seronegative Arthritis | None | Pembrolizumab | Yes | Yes | No | No |
| 35 | 58/M | MELANOMA | Cryoglobulinemia | Vitiligo | Nivolumab | No | No | No | No |
| 36 | 53/M | MELANOMA | None | None | Nivolumab | Yes | No | No | Yes |
| 37 | 54/M | BREAST | None | None | Atezolizumab | Yes | Yes | No | No |
| 38 | 78/M | MELANOMA | None | Colitis | Pembrolizumab | Yes | No | Yes | No |
| 39 | 72/M | LUNG | None | Colitis | Nivolumab | Yes | Yes | No | No |
| 40 | 77/M | LUNG | None | None | Nivolumab | Yes | Yes | No | No |
| 41 | 74/M | LUNG | None | None | Nivolumab | Yes | Yes | No | No |
| 42 | 63/F | LUNG | None | None | Nivolumab | Yes | Yes | No | No |
| 43 | 64/M | LUNG | None | None | Pembrolizumab | Yes | Yes | No | No |
| 44 | 76/M | LUNG | Gout | None | Pembrolizumab | Yes | Yes | No | No |
| 45 | 74/M | LUNG | None | None | Pembrolizumab | Yes | Yes | No | No |
| 46 | 62/F | LUNG | None | None | Pembrolizumab | Yes | Yes | No | No |
| 47 | 46/M | LUNG | None | None | Pembrolizumab | Yes | Yes | No | No |
| 48 | 66/M | RECTUM | None | None | Atezolizumab | Yes | Yes | No | No |
| 49 | 79/F | LUNG | None | None | Atezolizumab | Yes | Yes | No | No |
| 50 | 52/M | LUNG | None | None | Pembrolizumab | Yes | Yes | No | No |
| 51 | 58/F | RENAL | None | None | Pembrolizumab | Yes | No | No | No |
| 52 | 65/M | LUNG | Gout | None | Durvalumab | Yes | No | No | Yes |
| 53 | 85/M | RENAL | None | Hypothyroidism | Avelumab | Yes | No | No | Yes |
| 54 | 62/F | LUNG | None | None | Pembrolizumab | Yes | Yes | No | No |
| 55 | 60/F | LUNG | None | None | Nivolumab | Yes | Yes | No | No |
| 56 | 80/F | OVARIO | RA | None | Pembrolizumab | Yes | Yes | No | No |
| 57 | 73/F | THYROID | None | None | Durbalumab + Tremelimumab | Yes | No | No | No |
| 58 | 75/M | MYELODISPLASIC SYNDROME | None | None | Anti TIM3 | No | No | No | No |
| 59 | 77/M | RENAL | None | Colitis, hepatitis, Hypothyroidism | Ipilimumab+ Nivolumab | Yes | No | No | Yes |
| 60 | 75/M | LUNG | None | Pneumonitis | Durvalumab | Yes | No | No | Yes |
| 61 | 82/M | MELANOMA | None | Interstitial nephritis | Nivolumab | Yes | No | No | Yes |
| 62 | 72/M | LUNG | None | None | Durvalumab | Yes | No | No | Yes |
| 63 | 62/F | MELANOMA | None | None | Nivolumab | Yes | Yes | No | No |
| 64 | 74/M | ENT | None | None | Nivolumab | Yes | Yes | Yes | No |
| 65 | 76/M | UROTHELIAL BLADDER | None | None | Durvalumab | Yes | No | No | No |
| 66 | 88/M | MELANOMA | None | None | Pembrolizumab | Yes | No | Yes | No |
| 67 | 73/M | UROTHELIAL BLADDER | None | None | Ipilimumab+ Nivolumab | Yes | Yes | Yes | No |
| 68 | 46/M | RENAL CELLS CARCINOMA | None | None | Pembrolizumab | Yes | No | No | No |
| 69 | 55/M | LUNG | None | None | Pembrolizumab + Eftilagimod | Yes | No | No | No |
| 70 | 63/M | LUNG | None | None | Atezolizumab | Yes | Yes | Yes | No |
| 71 | 48/M | LUNG | None | Hypothyroidism | Nivolumab | Yes | Yes | No | No |
| 72 | 55/F | LIVER | None | Polyneuropathy | Durvalumab | No | No | No | No |
| 73 | 74/M | UROTHELIAL BLADDER | None | None | Ipilimumab + Nivolumab | Yes | Yes | No | No |

ENT: Otorhinolaryngology  RA: Rheumatoid arthritis, OA: Osteoarthritis, SLE: Systemic lupus erythematosus
