# Supplementary material for: Clinical Patterns and Follow-Up of Inflammatory Arthritis and Other Immune-Related Adverse Events Induced by Checkpoint Inhibitors. A Multicenter Study
Source: Front Med (Lausanne). 2022 Jun 15;9:888377. doi: 10.3389/fmed.2022.888377 (PMC9240301; doi:10.3389/fmed.2022.888377)
Supplement: Supplementary file 2 [file Table_2.docx]

**Supplementary Table 2. General characteristics of different series of patients with Rheumatic irAEs**

| **First Author (year) ^REF^** | **Location** | **No. of patients** | **Gender (Male)** | **Mean Age** | **Main**  **ICI** | **Oncologic Diagnosis** | **Other**  **irAE** | **Main Rheumatic syndromes** | **DMARD treatment** | **Auto antibodies** |
| --- | --- | --- | --- | --- | --- | --- | --- | --- | --- | --- |
| Calabrese et al (2017) ^2^ | Cleveland (USA) | 13 | 61% | 63.0 | Ipi+Nivo (46%)  Nivo (23%) | Melanoma (53%)  Renal cell carcinoma (30%) | Hypophysitis (30%)  Colitis (23%) | Inflammatory arthritis (53%)  Sicca syndrome (38%)  PMR -like (23%) | 30% | ANA 30%  RF 7.6% |
| Kostine et al (2018) ^3^ | Muticenter (France) | 35 | 65% | 63.8 | Anti PD-1/PD-L1 (85%)  Combined (14%) | Melanoma (45%)  Lung (34%)  Renal (17%) | Rash  Vitiligo  Psoriasis  Thyroiditis  Colitis | NIA (42%)  PMR-like (31%)  RA-like (20%) | 5.7% | ANA (11%)  CCP (2.8%) |
| Ritcher et al (2018) ^4^ | Rochester (USA) | 61 | 51% | 62,6 | Pembro (49%)  Nivo (21%)  Ipi (20%) | Melanoma (57%)  Lung (13%)  Lymphoma (10%) | Rash (34%)  Colitis (23%)  Intersticial  pneumonitis (8%) | Inflammatory arthritis (55%)  Others (27%)**  Myositis (16%) | 15% | NR |
| Mooradian et al (2018) ^5^ | Boston (USA) | 18 | 61% | 67.0 | PD-1/PD-L1 inhibitor (72%)  CTLA-4 (17%) | Melanoma (78%)  Lung (17%) | Colitis (31%)  Hypothyroid (19%)  Pneumonitis (13%) | Inflammatory arthritis (55%)  PMR-like (33%) | 50% | ANA 33%  RF 5%  CCP 0% |
| Capelli et al (2018) ^16^ | Baltimore (USA) | 27 | 55% | 60.2 | Anti PD-1 (66.6%)  Anti PD1+CTLA-4 (33.3%) | Melanoma (33.3%)  Lung (22.2%) | NA | Inflammatory arthritis | NR | RF 7.7%  CCP 7.7% |
| Arnaud et al (2018) ^32^ | Multicenter report | 86 | 53% | 63.5 | Nivo (53%)  Pembro (23%) | Lung (39%)  Melanoma (36%) | Muscle disorders (7%)  Colitis (5.8%) | RA -like  Autoimmune arthritis  Cutaneous lupus | NR | NR |
| Narvaez et al (2018) ^10^ | Barcelona (Spain) | 11 | 84% | 57.9 | Nivo (54%)  Pembro (18%)  Avelu (9%) | Lung (36%)  Lymphoma (18%)  Melanoma (9%) | NR | Inflammatory arthritis (45%)  Myositis (18%)  PMR-Like (1%) | 18% | ANA 27%  RF (0%)  CCP (0%) |
| Tison et al (2019) ^34^ | Multicenter (France) | 112* | 57 | 66.5 | Nivo (56%)  Pembro (24%)  Ipi (13%) | Melanoma (58%)  Lung (35%)  Urothelial (3.5%) | Vitiligo (7.1%)  Colitis (6.2%)  Thyroiditis (5.3%) | Arthralgia  Inflammatory arthritis  Shoulder tendinitis | 21%* | NA |
| Braaten et al (2020) ^24^ | Baltimore (USA) | 60 | 46% | 58.5 | Monotherapy (70%)  Combination therapy (30%) | Melanoma (35%)  Lung (23%)  GI (11%) | Rash (33%)  Colitis (33%)  Thryoiditis (26%) | Inflammatory arthritis | 31% | ANA 14%  RF 1.8%  CCP 5% |
| Roberts et al (2020) ^26^ | Multicenter (Canada) | 117 | 59% | 62.3 | Pembro (31%)  Nivo (25%) | Melanoma (48%)  Lung (25%) | Rash (17%)  Psoriasis (13%)  Colitis (13% | Symetric polyarthritis (33%)  PMR-like (12.5%) | 35% | ANA 17%  RF 9.3  CCP 4.5% |
| Kim ST et al (2022)^29^ | Texas (USA) | 20 | 70% | 56.5 | Nivo (30%)  Ipi +Nivo (30%)  Pembro (20%)  Durva (5%) | Melanoma (55%)  Lung (20%)  Renal cell carcinoma (20%) | Colitis (35%)  Dermatitis (20%) | Undifferentiated oligo (65%)  Undifferentiated poly (20%)  Monoarthritis (15) | 45% | NA |
| Current series | Barcelona (Spain) | 73 | 64% | 66.1 | Pembro (32%)  Nivo (23%)  Atezo (9%) | Lung (39%)  Melanoma (27%)  Renal-urothelial (15%) | Colitis (8%)  Hyothyroidism (4%)  Polyneuropathy (2%) | RA-like (42%)  NIA (26%)  PMR-like (9%)  PsA-like (6%) | 11% | ANA (33%), RF (6.8%), CCP (5.6%) |

ANA: Antinuclear antibodies, Atezo: Atezolizumab, Avelu: Avelumab, CCP: Anti-Cyclic citrullinated proteins antibodies Ipi: Ipilimumab, Pembro: Pembrolizumab, PMR: Polymyalgia rheumatica, NA: Not available, NIA:Non-inflammatory arthralgias, Nivo: Nivolumab, NR: Not reported, RA: Rheumatoid arthritis, RF: Rheumatoid factor

% Reported when available

*Cohort of patients with preexisting autoimmune disease

** Others: vasculitis, PMR-like syndrome, CTD, or flare of preexisting rheumatic disease
